# Effects and Pain Levels in Nulliparous Pregnant Women Undergoing Cervical Ripening With the Controlled-Release Dinoprostone Vaginal Delivery System (PROPESS) Compared With Mechanical Methods

**DOI:** 10.7759/cureus.101216

**Published:** 2026-01-10

**Authors:** Shunji Suzuki, Nobuko Yokoyama

**Affiliations:** 1 Obstetrics and Gynecology, Japanese Red Cross Tokyo Katsushika Perinatal Center, Tokyo, JPN

**Keywords:** cervical ripening, controlled-release dinoprostone vaginal delivery system (propess), mechanical methods, numeric rating scale, pain level

## Abstract

Objective

We compared the effects of controlled-release dinoprostone vaginal delivery system (PROPESS; Ferring Pharmaceuticals, Saint-Prex, Switzerland) use on cervical ripening and on pain levels, compared with mechanical methods.

Methods

The criteria for inclusion in this study were as follows: nulliparity, singleton pregnancy, cephalic presentation, gestational age of 41 weeks, and a Bishop score at the start of induction equal to or less than 4. During the study period, 25 women used PROPESS, and 25 women used mechanical methods for cervical ripening at 41 weeks of gestation. After the selected insertion methods were completed, the patients were asked about their pain levels during the insertion, with the pain scored on a 10-point numerical pain-rating scale (Numeric Rating Scale, NRS). Successful cervical ripening was defined as a Bishop score >6 at removal, or if the patient delivered vaginally by the next day following insertion of PROPESS only or mechanical cervical dilation only.

Results

There were no significant differences in the rate of unsuccessful cervical ripening cases between the two groups; however, the average NRS score at PROPESS insertion was significantly lower than that during mechanical methods.

Conclusions

The cervical ripening effect of PROPESS was not significantly different from that of mechanical methods in women requiring cervical ripening. PROPESS also had the potential benefit of being pain-free during cervical ripening.

## Introduction

Controlled-release dinoprostone vaginal delivery system (PROPESS; Ferring Pharmaceuticals, Saint-Prex, Switzerland) has been approved since 2020 in Japan as a new cervical ripening therapy for pregnant women at term prior to labor induction [1]. To date, some studies have been performed considering the effectiveness or pain levels in cases using PROPESS, compared with mechanical methods for cervical ripening [2-4], because the disadvantages associated with pain prior to labor induction should be considered as important as the benefits of cervical ripening in clinical practice [4-8]. However, previous studies have examined efficacy and pain separately, and no reports integrating these aspects have been observed [2-4]. Some researchers have indicated that both options - ripening with PROPESS and with mechanical methods - are similarly effective [2-4,9,10], and combining them can be successful [3]. On the other hand, in our earlier pilot study, the pain scored on a 10-point Numeric Rating Scale (NRS) with PROPESS insertion was significantly lower than that with mechanical methods [4].

Based on this background, we compared the effects of PROPESS use on cervical ripening and pain levels, compared with mechanical methods.

## Materials and methods

This study was approved by the Ethics Committee of the Japanese Red Cross Tokyo Katsushika Perinatal Center, Tokyo, Japan (approval no. K-23-011), and all participants gave informed consent prior to their inclusion.

This prospective cohort study was performed at the Japanese Red Cross Katsushika Perinatal Center, one of the main perinatal centers in Tokyo, Japan, between December 2023 and June 2024. The criteria for inclusion in this study were as follows: nulliparity, singleton pregnancy, cephalic presentation, gestational age of 41 weeks, and a Bishop score at the start of induction equal to or less than 4. These inductions of labor were performed to prevent post-term pregnancy. The exclusion criteria in this study were perinatal complications such as premature rupture of the membranes (PROMs), hypertensive disorders, and oligohydramnios. Pregnant women assessed as having a mental disorder or unstable mental state were also excluded, because, based on our previous study, the subsequent assessment of pain levels as a clinical trial could potentially be traumatic for them [4].

During the study period, 25 women used PROPESS, and 25 women used mechanical methods for cervical ripening at 41 weeks of gestation. Clinical data, including maternal characteristics, were obtained from hospital records.

During the study period, when a Bishop score ≤4 was observed prior to labor induction without PROM at 41 weeks of gestation, PROPESS or mechanical methods (insertion of laminaria tents or their synthetic equivalent, such as Dilapan, in the cervical canal) were selected freely as suitable intervention options [4]. In cases of PROPESS insertion, PROPESS was held between the second and third fingers while wearing medical gloves, inserted into the posterior vaginal canal, and retained in the vaginal canal. On the other hand, in cases of Dilapan insertion, a vaginal speculum was inserted, and the vaginal canal was washed with a saline solution. The cervix was then held with a single hook forceps, and one to five pieces of Dilapan, pinched with the forceps, were inserted into the cervical canal slowly and held in place with a gauze moistened with saline solution to inflate the Dilapan.

In this study, the number of implementers was limited to one in order to eliminate differences between implementers, and the number of Dilapan insertions was limited to a maximum of five pieces, avoiding attempted force insertion. After the selected insertion methods were completed, the patients were asked about their pain levels during the insertion, with the pain scored on a 10-point numerical pain-rating scale (NRS), a simple and commonly used tool for measuring pain intensity from 0 to 10 [4,11]. A score of 0 represents “no pain,” and 10 represents the “most intense pain possible.” Patients were asked to rate their level of pain by choosing a number on the scale that best represented their pain intensity. The interviews about pain were conducted during fetal heart rate monitoring, approximately 20-60 minutes after the insertion, and patients were asked to rate the pain they felt most strongly during the insertion.

To evaluate the effects of PROPESS and mechanical methods, we examined the Bishop score at removal. Successful cervical ripening was defined as a Bishop score >6 at removal [1], or if the patient delivered vaginally by the next day following insertion of PROPESS only or mechanical cervical dilation only. Cases that did not meet these criteria were defined as unsuccessful cervical ripening cases.

Data are presented as numbers (percentages) or mean ± standard deviation (SD). Statistical analyses were performed with SAS version 8.02 (SAS Institute, Cary, NC, USA). Cases were compared using the Student’s t-test for continuous variables, or the Chi-square test or Fisher’s exact test for categorical variables. Statistical significance was set at p < 0.05.

## Results

Table 1 shows the clinical characteristics of the women in the PROPESS and mechanical-method groups. There were no significant differences in any variables between the two groups.

**Table 1:** Clinical characteristics of women in the PROPESS (controlled-release dinoprostone vaginal delivery system) and mechanical-method groups Data are presented as numbers (percentages) or mean ± standard deviation (SD).

	Mechanical Methods Group (Total, n = 25)	PROPESS Group (Total, n = 25)	T-value	p-value
Maternal age (y)	34 ± 4	34 ± 4	0.354	0.9
Maternal height (cm)	157 ± 8	156 ± 7	1.19	0.29
Maternal BMI at admission	27.8 ± 3.3	28.1 ± 3.7	-0.567	0.57
Bishop score at admission	3.3 ± 0.7	3.6 ± 0.6	-1.21	0.19
Neonatal birth weight (g)	3098 ± 412	3138 ± 399	-2.02	0.12

Table 2 shows the NRS score at PROPESS insertion or during mechanical methods, the Bishop score at the removal of PROPESS or Dilapan, and the number of cases without successful cervical ripening. There were no significant differences in the rate of unsuccessful cervical ripening cases between the two groups; however, the average NRS score at PROPESS insertion was significantly lower than that during mechanical methods (p < 0.01).

**Table 2:** Numeric Rating Scale score at cervical ripening procedures with PROPESS (controlled-release dinoprostone vaginal delivery system) or Dilapan insertion, Bishop score at the removal of PROPESS or Dilapan, and the number of cases without successful cervical ripening in the study Data are presented as numbers (percentages) or mean ± standard deviation (SD).

	Mechanical Methods Group (Total, n = 25)	PROPESS Group (Total, n = 25)	F-value/ T-value	Degree of Freedom	Phi Coefficient	p-value
Numeric Rating Scale score at cervical ripening procedures	5.8 ± 3	2.5 ± 2	7.38	-	-	<0.01
Delivery by the next day	2 (8)	3 (12)	0.222	1	-0.067	0.64
Number of cases without delivery by the next day	23 (92)	22 (88)	0.222	1	-0.067	0.64
Bishop score at the removal	5.9 ± 2.2	6.4 ± 2.9	-1.11	-	-	0.30
Unsuccessful cervical ripening cases	13 (52)	9 (36)	1.30	1	0.161	0.25

The average NRS score at PROPESS insertion in the successful cervical ripening cases was 2.7 ± 2, which was not different from that in the unsuccessful cervical ripening cases (2.3 ± 2, p = 0.49). In addition, there was no significant difference in the average NRS score during mechanical methods between the successful and unsuccessful cervical ripening cases (5.9 ± 3 vs. 5.3 ± 3, p = 0.41).

## Discussion

The current results support previous observations indicating the similar effectiveness of PROPESS and mechanical methods for cervical ripening in pregnant women requiring labor induction at term [2,3]. In addition, the results support the potential pain-free benefit of PROPESS compared with mechanical methods during cervical ripening [4]. Therefore, choosing a painless method for cervical ripening, such as PROPESS, may lead to a more comfortable delivery and better postpartum condition for patients. These benefits are expected to reduce the incidence of perinatal mental health problems, such as postpartum depression, which have been on the rise worldwide in recent years [5-8,12]. This is still a small-scale study; however, we believe that the effects of PROPESS are supported by statistical significance.

In this study, no significant correlation was observed between cervical ripening effects and pain levels in either group. We had hoped that the effect on the degree of cervical ripening could be predicted by pain levels during the procedures; however, the expectation was not met. Considering that the mechanism of pain during the procedures is related to various factors, including the mental state of the pregnant woman prior to labor induction, the effect of poor cervical ripening itself on pain levels may not be major.

In Japan, although the rate of painless delivery using epidural anesthesia has been increasing later than in other developed countries [13], it may be difficult to initiate effective pain relief during the cervical ripening phase; therefore, the spread of such pain-free cervical ripening methods is considered clinically very effective for pregnant women requiring induction of labor. In Japan, where nulliparous women of advanced age are particularly increasing, such methods are likely to become increasingly necessary.

Finally, we cannot deny that there are some serious limitations, apart from participant fear, considering that this was not a large-scale study. Firstly, in this study, the effectiveness of the two methods was evaluated using the difference in changes in Bishop score; however, potential ripening that does not manifest in the Bishop score was not assessed [14,15]. Cervical ripening related to the response to labor-inducing drugs could not be evaluated. Since the two cervical ripening methods differed greatly in economic cost, the choice of method was left to the participants’ discretion rather than being randomly assigned, so it cannot be ruled out that bias may have occurred in the selection of ripening methods.

## Conclusions

In this study, we compared the effects and pain levels of PROPESS on cervical ripening with those of mechanical methods in pregnant women prior to labor induction at 41 weeks of gestation. The cervical ripening effect of PROPESS was not significantly different from that of mechanical methods; however, the average NRS score at PROPESS insertion was significantly lower than that during mechanical methods. Therefore, PROPESS had the potential benefit of being pain-free during cervical ripening.
